# Some Cases Illustrative of the Nature and Treatment of Pain about the Face—Facial Neuralgia

**Published:** 1860-10

**Authors:** C. McElroy


### ARTICLE IX.

*Some Cases Illustrative of the Nature and Treatment of Pain about the Face—Facial Neuralgia.* By C. McELROY, M. D.

No attempt will be made in this paper to present an elaborate monograph on the subject of facial neuralgia, or pain about the face—but, some cases will be narrated of very opposite pathological character, and relieved by very different remedial measures.

The reader will understand, then, that what follows are the actual experiences of a country physician, with the realities narrated in a style of a professional conversation, seizing the distinguishing features, rather than the minutia of their progress, to recovery.

W. F., æt. between 25 and 30, is a laboring man, of ordinary good health, a teamster, and married. When exposed to the chilly wet weather of early autumn and spring, is frequently seized with face-ache, commonly confined to one side, with very tender teeth—many of them carious—but still only giving trouble on such occasions. This state of things yields very kindly to one or two decisive doses of morphine, followed, after the subsidence of the pain and tenderness, by a saline or oily purge. He seldom loses a day, if the decisive doses of morphine are taken at night.

A peculiarity of his case is, that no other form of opiate produces this result. This case is classified as one of facial neuralgia strictly.

Mrs. J. B. A., is a lady in the meridian of life, good general health, with the cares of a large family pressing on her; became troubled with a cough in the autumn of 1859—probably a sub-acute bronchitis—and possibly, somewhat asthmatic, which excited but little solicitude. Domestic, or homeopathic treatment only, was made use of, up to the early part of January, 1860, when opportunity offering, she made a trip of some hundreds of miles to join in some festivities in the family of a relative; her family thinking the trip would benefit her.

Contrary to their and her expectations, she became much worse during her outward bound trip, and arriving at her destination, her powers of endurance were sorely taxed to join in the festivities for which she had gone. She continued to grow worse until she arrived at home again, having been absent about a week. A homeopathic physician saw and prescribed for her during the next three days, but she continued steadily to grow worse. Saw this lady in the forenoon of Tuesday; found her with extremely anxious countenance, flushed face, rapid pulse; considerable cough; intense pain in her right eye and about the temple; great intolerance of light; tongue furred, appetite completely gone, and sense of extreme exhaustion. A sad picture indeed. Her only entreaty was relief from the agonizing pain about the eye and in the eye, or she would go blind.

With as little delay as the importance of the case would admit, an emetic was decided upon as the first step in the treatment. Ipecac. and capsicum. After emesis to take hyd: c. creta, Dover pow. and morphine; chloroform and heat to her eye and temple.

Wednesday.—Emetic had operated freely. Had taken powders as directed. Stomach had been more or less irritable all night, with occasional vomiting, and on the whole very little better. Pain intermits occasionally. Treatment to-day, brandy and morphine; mint water, with creta ppt., to settle stomach. Chloroform and aconite to temples, with heated flannels, or raw cotton to the eye and temple. To take some nourishment if possible.

Thursday.—Patient very little better. Pain very severe. Could not take brandy—thought it made her worse. Had a very bad night. Is suffering severely to-day. Applied magnets—electricity, with no relief. Mustard poultices to the stomach; hot foot baths; milk punch; morph. and hyd: c. creta, with a continuation of the hot applications to eye and temple.

Friday.—Patient no better—passed a bad night again. Pain about temple and eye still very intense. No motion of bowels. The existing state of things was very discouraging both to patient and physician, and her urgent cry was still for relief from the intense pain. Asked for ice, salt and thin cloth. Pounded the ice fine, and proceeded to make a freezing mixture to apply to the seat of the pain. Patient very much alarmed, as was her daughter; wanted the matter postponed; was certain it would make her worse. When all was ready, permission was obtained to try it. The pounded ice and salt, enveloped in a thin cloth, was applied and kept on, perhaps, half a minute; patient complaining of intense burning. A little rest and it was re-applied, and removed as often as she desired, during a quarter of an hour. When the application was over, all pain and soreness was completely gone, and patient was very much relieved, and expressed herself very grateful for it. Directed ice and salt again, if pain should return—to take a seidlitz powder, and eat something.

Did not expect to visit patient again without being sent for, and on Saturday morning had a message from the family to the effect that the medicine had moved her bowels so severely that she was in great danger from exhaustion. Hurried off to see her; (three miles.) Found her entirely relieved of all pain and soreness about eye, tongue cleaning, pulse good, but somewhat feeble, and every thing in the best possible way. Gave a little brandy and creta ppt., and requested its repetition in case her bowels continued to act, and to eat as much as she could.

The relief was perfect and complete. In less than a week she was quite well, and had regained nearly all her loss.

This was a very curious and interesting case to me. Its pathology is not very plain. The most probable hypothesis is, that the nerves or nerve coverings about the temple and eye, were in a state of inflammation or irritation. One thing is certain, that the narcotics externally and internally, did not benefit her much, if any. The freezing mixture was most likely the potential counter-irritant, combined with the mercurial alterative, that removed the difficulty. The physician was quite as much gratified as the patient at the somewhat unexpected, sudden, and perfect recovery of the patient, from so harrassing and distressing a difficulty.

A. L. W., æt. 35, an active though nervous gentleman, enjoying in the main good health, is at uncertain times seized with violent pains in the right eye, which soon becomes intolerant of light, and deeply infected with blood, accompanied by rapid pulse, flushed face, anxious countenance, &c. Is generally relieved by a decisive dose of morphine and calomel, and not by either separately, as found by frequent experiment. Upon one occasion, several years since, had to have a slightly carious tooth removed before recovering, after vainly striving with remedial agents two weeks. After the removal of the tooth, his recovery was rapid and complete.

Mrs. J. B., a lady eighty years of age, has been remarkably healthy during her long life, entered the summer of 1860 with crippled health, tongue furred, appetite bad, with great sense of exhaustion and weakness, but apparently no settled disease, unless a rheumatic condition of the lower extremities could be so called. The limbs were not in actual pain, but stiffened, and difficult to move. All this stiffness upon a sudden, took its leave, and in its place she had a violent ache in the head and face. Diagnosing this to be a metastasis of her rheumatism, every effort was made to repel it from the head, and invite it back again to her feet and limbs, chloroform, aconite, laudanum, camphor, ammonia, and the freezing mixture, were in turns applied to her head and face with but little relief; while stimulant foot baths, frictions and poultices, were applied to the extremities. She bore opium very badly, and its use was, consequently limited, but she had brandy and narcotics. Small doses of calomel with an occasional dose of Husband's magnesia, were the effective agents in her relief. She is now rapidly regaining health and strength.

Mrs. J. S. C., an exceedingly delicately organized lady, in the meridian of life, whose residence is in New York city, with, for the last four or five years, fair average health; was taken ill while on a visit to relatives in our city, during the last summer.

From her peculiar organization she had some idiosyncrasies in regard to standard remedies which forbid, as she had been told in New York, their use in her case. I found opium was out of the question, and she had a great terror of calomel and blue pill.

Respecting, as I did, her prejudices, they were not employed in the earlier part of the treatment of her sickness, though indicated at times very strongly. Her case was diagnosed as *hay asthma* or *autumnal catarrh*, characterised by severe cough, great sense of exhaustion, intensely reddened mucous membranes; and at a later stage, intense pain in the head. Finding it impossible to obtain relief from stimulants and external applications, recourse was had to an emetic, followed by blue pill and magnesia, with prompt and effectual relief, as she rapidly recuperated, recovered strength and appetite, and went home very much improved, with few, if any, traces of her tedious illness while absent.

J. T., æt. 25; cotton factor, of good general health; consulted me in the fall of 1859, for intermittent headache, for which quinine was prescribed, which, however failed to arrest it. The doses were repeated in much larger quantities until it was satisfactorily ascertained that they would not arrest it. Local applications of every conceivable sort, including electricity, also failed. Alteratives, with purging remedies, were also prescribed, with like results. The last prescription was carb. ferri. precip: which likewise failed, when he ceased to consult me, having made up his mind he could not get rid of it. Some time after, a tooth-ache sent him to a dentist, who extracted it, after which, for months, his headache never returned. It did however return, when a tooth was found tender and extracted, since which, up to this time, he has had no return of the pain in his head, and enjoys very good health.

No claim is made to any of the cases cited, as being, in themselves, very remarkable; on the contrary, they are common; but they fairly represent several aspects of facial neuralgia, and pain in the head. From them we may deduce as follows:

That the cause of pain in the face, or about the head, in the majority of cases, must be sought for elsewhere, than the seat of the pain.

That local applications, of themselves, do not possess much value as remedial agents. The apparent exception in the second case, must be received with some caution, as the constitutional treatment was in progress at the time of the application of the freezing mixture, and culminated in the critical discharges from the bowels the following afternoon and night; though due credit must be given for the prompt relief it gave at the time.

That the general principles applicable to the treatment of inflammation, irritation and congestion, are pretty certain to bring relief of a permanent character, as opposed to the plan of temporary relief from external applications, and stimulants internally.

That when the cause is located in a caries of the teeth, or periosteal inflammation, constitutional treatment, for the most part fails; the tooth or teeth must sooner or later be removed—of this character was the third and last case.

That where the irritation is in the nerve of the decayed tooth or teeth alone, decided doses of narcotic stimulants will, for the most part, be successful.

Lastly,—That while, as physicians, we must respect the prejudices and opinions of patients, we must not allow them to interfere too long with correct medical diagnosis and theapeutics, based upon well ascertained truths in the healing art; for, at a proper moment, the prejudices of all sufferers will give way to correct and rational treatment, founded in general principles, and with a well grounded hope of relief.

